# A high interferon gamma signature of CD8^+^ T cells predicts response to neoadjuvant immunotherapy plus chemotherapy in gastric cancer

**DOI:** 10.3389/fimmu.2022.1056144

**Published:** 2023-01-05

**Authors:** Sen Li, Ke Li, Fei Tian, Hongle Li, Qingxin Xia, Tiepeng Li, Bing Dong, Danyang Li, Juan Yu, Junli Zhang, Li Wang, Chengjuan Zhang, Shuning Xu, Yuzhou Zhao, Ying Liu

**Affiliations:** ^1^ Department of General Surgery, Affiliated Cancer Hospital of Zhengzhou University, Henan Cancer Hospital, Zhengzhou, Henan, China; ^2^ Department of Medical Oncology, Affiliated Cancer Hospital of Zhengzhou University, Henan Cancer Hospital, Zhengzhou, Henan, China; ^3^ Department of Biological Information, Genesky Biotechnologies Inc., Shanghai, China; ^4^ Department of Molecular Pathology, Affiliated Cancer Hospital of Zhengzhou University, Henan Cancer Hospital, Zhengzhou, Henan, China; ^5^ Department of Pathology, Affiliated Cancer Hospital of Zhengzhou University, Henan Cancer Hospital, Zhengzhou, Henan, China; ^6^ Department of Immunotherapy, Affiliated Cancer Hospital of Zhengzhou University, Henan Cancer Hospital, Zhengzhou, Henan, China; ^7^ Department of Endoscopy Center, Affiliated Cancer Hospital of Zhengzhou University, Henan Cancer Hospital, Zhengzhou, Henan, China

**Keywords:** gastric cancer, single-cell RNA sequencing, tumor microenvironment, interferon gamma, immune checkpoint blockade, neoadjuvant immunotherapy

## Abstract

**Background:**

While the tumor microenvironment (TME) affects immune checkpoint blockade (ICB) efficacy, ICB also reshapes the characteristics of TME. Thus far, studies have focused on the TME evolution during neoadjuvant or adjuvant ICB therapy in gastric cancer (GC). However, the interaction between TME characteristics and neoadjuvant immunotherapy plus chemotherapy remains to be elucidated.

**Methods:**

We performed single-cell RNA sequencing on ten GC specimens pre- and post-neoadjuvant camrelizumab plus mFOLFOX6 to determine the impact of the TME on the efficacy of the combination therapy and the remodeling of TME by the therapy.

**Results:**

A high baseline interferon gamma (IFN-γ) signature in CD8^+^ T cells predicts better responses to the combination therapy. We also observed that the IFN-γ signature significantly decreased in multiple cell types, and the exhausted signature of CD8^+^ T cells was significantly suppressed during the neoadjuvant therapy.

**Conclusions:**

Our data reveal interactions between the TME and neoadjuvant immunotherapy plus chemotherapy in GC. Importantly, it also highlights the signature of CD8^+^ T cells in predicting response to the combination therapy in GC.

## Introduction

Gastric cancer (GC) is a highly aggressive malignant tumor, ranked fifth for incidence and fourth for mortality with an estimated 769,000 deaths globally in 2020 (1). Early-stage GC is associated with a 5-year survival rate of ~95%, whereas, patients with advanced/metastatic GC have a median survival of 9-10 months (2). Immune checkpoint blockade (ICB) has paved the way to a new era of GC immunotherapy (2–4). The phase III checkmate 649 trial demonstrated that nivolumab plus chemotherapy compared to chemotherapy as first-line treatment improved the overall survival (OS) and progression free survival (PFS) of patients with advanced gastric or gastro-oesophageal junction (GC/GEJ) adenocarcinoma cancer, whether the PD-L1 positive score was more than 5 or not (5). Due to the achievements of immunotherapy in advanced GC/GEJ adenocarcinoma cancer, more studies have begun to explore the neoadjuvant therapy at present. However, not all GC patients respond to neoadjuvant ICB. Therefore, there is a need to explore underlying mechanisms and associated markers to screen GC patients who might benefit from immunotherapy.

The tumor microenvironment (TME) plays an important role in the response of gastic cancer to immunotherapy (6). Previous studies have explored the TME features using bulk sample-based experiments or mathematical models (7–9). However, the TME of GC is complex and heterogeneous, and the bulk sample-based experiments obscure the signatures of distinct cell populations. Therefore, it is necessary to elucidate the molecular and cellular landscape, their dynamics, and functional characteristics of TME in GC patients with ICB therapy.

In recent years, high-throughput single-cell RNA sequencing (scRNA-seq) opened a new way for dissecting heterogeneous tumors and deciphering the transcriptional features betweencancer cells, microenvironment components, and their interactions (10–12). In GC, scRNA-seq has been utilized to characterize the transcriptional heterogeneity of gastric tumoral and normal tissues (13–16). Wang et al. reported on the intratumoral diversity of metastatic GC by scRNA-seq profiling of peritoneal carcinomatosis and identified a new prognostic gene signature related to tumor cell lineage and state compositions (17). Kim et al. revealed the early remodeling of the TME in patients with advanced gastric cancer during first-line chemotherapy by scRNA-seq. Response to chemotherapy in advanced GC was associated with on-treatment TME remodeling including NK-cell recruitment, decreased tumor-associated macrophages, M1-macrophage repolarization, and increased effector T-cell infiltration (18). Unfortunately, few studies focus on the cellular diversity and dynamic changes of TME in response to ICB therapy in GC.

In this study, we performed scRNA-seq of 5 paired tumor specimens pre-treated and on-treatment with 4 cycles of chemotherapy plus anti-PD-1 antibody (camrelizumab) as neoadjuvant therapy in locally advanced GC/GEJ. We showed inter- and intra-tumor cellular heterogeneity and transcriptional changes of diverse cell types during the neoadjuvant therapy. An IFN-γ signature was enriched in pre-treated tumors; specifically, a high IFN-γ signature in CD8^+^ T cells correlated to positive response to the combined therapy This study sheds light on transcriptional dynamics at the single-cell level within GC during immunochemotherapy and provides new insights for the use of neoadjuvant ICB in GC.

## Materials and methods

### GC patients and clinical study

Patients were pathologically diagnosed with GC/GEJC at Zhengzhou University Affiliate Cancer Hospital and enrolled in the prospective single-arm, phase 2 study of Camrelizumab combined with mFOLFOX6 as neoadjuvant therapy for resectable, locally advanced GC/CEJC (clinical trial information: NCT03939962). They received 4 cycles of mFOLFOX6 plus anti-PD-1 antibody (camrelizumab) as neoadjuvant treatment followed by a gastrectomy with D2 lymph node dissection. All patients gave informed consent for collection of clinical information and tumor tissue for research testing. Five patients with mismatch repair protein proficient (pMMR), or microsatellite stable (MSS), that underwent a gastrectomy with D2 lymph node dissection were selected randomly. Pre- and on-treatment paired tumor samples of the five patients were collected for following scRNA-seq.

### Preparation of single-cell suspensions

Fresh tumor tissue samples were obtained from 5 GC patients by endoscopic ultrasonography-guided biopsy from surgery before and after treatment. Tissue was immediately immersed in RPMI 1640 medium (Thermo, 11875-085) for subsequent single-cell isolation. Tissue dissociation was performed with Tumor Dissociation Kit (Miltenyi Biotec, 130-095-929) at 37°C for 30-45 min and filtered using a 40μm cell strainer (BD, 352340). Erythrocytes were removed by RBC lysis buffer (Solarbio, R1010). Cell suspensions were washed 2 times with PBS containing 0.04% BSA at 300g for 5 min at 4°C. Cell viability was measured with Trypan Blue (Thermo fisher, 15250061) staining. Cell concentration was measured with hemocytometer and adjusted to 700-1,500 cells/μL.

### Single-cell capture, library preparation and sequencing

The single-cell suspensions were then subjected to single-cell capture using the Chromium platform (10× Genomics). Chromium platform is a droplet-based system in which GC single cells, gel beads with barcoded oligos, and reagents were mixed and captured as droplets in oil emulsion. Single-cell library preparation was performed using 10× Genomics Single Cell 3’ Reagent v3 Kit according to the manufacturer’s instructions. Library quality was assessed with Agilent 2100 Bioanalyzer (Agilent). Libraries were pooled and sequenced on the Illumina HiSeq X Ten platform (Illumina) and generated at least 50K 150bp pair-end reads per cell.

### Single-cell RNA-seq data preprocessing and clustering of major cell types

The CellRanger software package (version 3.1.0) was adopted to process the 10× Genomics raw data based on the human reference genome GRCh38. Raw gene expression matrices were analyzed using Seurat R package (version 3.1.4) with the following criteria for cell filtering: (1) all cells expressing lower than 200 or larger than 6000 genes were removed; (2) cells containing 50% or more of UMIs mapped to mitochondrial or ribosomal genes were eliminated if they met one of the standards. With the remaining 35,884 cells, gene expression matrices were normalized and subsequently dimensionally reduced based on 2,000 highly variable genes detected by the “FindVariableGenes” function. For the clustering of major cell types, the top 50 principal components were selected with a resolution parameter equal to 0.8. Finally, major cell clusters projected in the two-dimensional Uniform Manifold Approximation and Projection (UMAP) representation were annotated to known cell types using well-recognized marker genes.

### Re-clustering of immune and non-immune cell populations

To identify sub-populations within immune and non-immune cell clusters from pre- and on-treatment samples, cells were extracted *via* the “SubsetData” function. Then, we re-clustered the selected cells by the second-round UMAP reduction. The number of principal components in each subtype was independently determined by the “Elbowplot” function implemented in Seurat. The sub-clusters were annotated by the dominantly expressed cell markers in previous studies (19–21).

### Identification of marker genes of cell sub-clusters

To identify marker genes for each sub-cluster within T cells or other cell types, the “FindAllMarkers” function in Seurat was used to compare cells of the studied sub-cluster with all other sub-clusters of this cell type. Marker genes of sub-clusters were defined as having a threshold fold change > 0.25 in the studied sub-cluster compared to the other sub-clusters and with detectable expression in > 25% of the cells in that sub-cluster. Additionally, marker genes were required to have the highest mean expression in the studied sub-cluster compared to all other sub-clusters.

### RNA sequencing and analysis in bulk tumor tissues

Pre- and on-treatment paired tumor tissue samples from 14 GC patients in our cohort were obtained for bulk RNA sequencing. Total RNA was extracted from tumor samples using TRIzol LS reagent (Thermo) and quatified by Agilent 2100 Bioanalyzer (Agilent). The ribosomal RNA was depleted by Ribo-Zero™ rRNA Removal Kit (Illumina). Finally, the mRNA sequencing library was constructed according to the protocols of TruSeq Stranded Total RNA Library Prep Kit (Illumina). RNA sequencing was performed with paired-end 2 × 150bp on Illumina Hiseq X Ten platform (Illumina). The clean reads of mRNA were aligned to the human reference genome GRCh38 using HISAT2.

### Cell-type infiltration analysis in bulk tissue based on scRNA-seq data

To estimate the proportions of our defined major cell populations and subpopulations in bulk RNA-seq data, we used the online tool CIBERSORTx to create a reference signature matrix from our scRNA-seq data (22).

### Differential expression and pathway analysis

Differentially expressed genes (DEGs) between different cells or time courses were identified using the “FindMarkers” functions in Seurat with a threshold for logFC > 0.25 and expression in a minimum fraction of cells > 25%. The R package hypeR (v1.8.0) and the Hallmark gene sets were used for pathway analysis on DEGs of different groups.

### Definition of cell scores and signatures

We used the average expression levels (measured by log_2_ (normalized counts)) of 7 cytotoxicity associated genes (PRF1, IFNG, GNLY, NKG7, GZMB, GZMA, CST7 and TNFSF10) and 5 exhausted markers (CTLA4, HAVCR2, LAG3, PDCD1 and TIGIT) to define the cytotoxic and exhausted scores for CD8^+^ and CD4^+^ T cells (19). The IFN-γ and expanded immune signature were defined by 6 and 18 genes, respectively (23). To calculate the M1/M2 polarization and anti-/pro-tumor potential of macrophage cells, M1 or M2-associated genes were used to define the signature of macrophages (24, 25).

### Trajectory analysis of CD8^+^ T cells

We performed the trajectory analysis *via* Monocle 2 (version 2.20.0) (26) to explore the effect of treatment on CD8^+^ T cell in our scRNA-seq data. First, the function “newCellDataSet” was applied to construct the Monocle subject. The gene signatures used to annotate the Monocle components are the marker genes of sub-clusters within CD8^+^ T cells. The “reduceDimension” function was applied to reduce dimensions, and we placed cells onto the pseudotime trajectory with default parameters by “orderCells” functions. The phase gene sets of CD8^+^ T cell trajectory were enriched by gene set enrichment analysis (GSEA) with the Hallmark and Reactome pathway.

### Copy number analysis of epithelium

To further distinguish malignant and non-malignant epithelial cells, we calculated CNVs in epithelial cells based on our scRNA-seq data using R package inferCNV (version 1.6.0) as described previously (27). T cells served as the background to calculate the CNV score of epithelium. This package compared the expression intensities of genes across epithelium and related this to expression in T cells. The k-means clustering was used to exhibit possible non-malignant cells which had similar modified expression levels with T cells.

### Constructing cell interaction network *via* CellPhoneDB

CellPhoneDB Python package (2.1.7) (28) used the cluster annotation and counts from our scRNA-seq data to compute cell-cell communication between CD8^+^ T cells and other cells (epithelium and macrophages) pre- and post-treatment. The default ligand-receptor pair information was used in this process. A p value ≤ 0.05 indicated significant enrichment of the interacting ligand-receptor pair in each interacting pair of cell subtypes, and only receptors and ligands expressed in more than 10% of the cells in the interacting subpopulations were considered. Log_2_ mean referred to the log_2_-transformed total mean of the individual partner average expression values in the corresponding interacting pairs of cell subtypes.

### Survival analysis

The Kaplan-Meier Plotter analysis tool (http://kmplot.com/analysis/) was used to access the association between gene expression status and prognosis in GC patients. The Kaplan-Meier Plotter database incorporated multiple GEO datasets and TCGA GC cohort for predicting prognosis (29).

### Statistical analysis

All data processing was performed using R 3.6.1 software. The statistical tools, methods, and thresholds for each analysis are explicitly described in Materials and Methods or detailed in the Figure legends. All statistical results with a p-value < 0.05 were considered to indicate statistical significance.

## Results

### Single-cell transcriptome profiling of GC tumors during the neoadjuvant therapy

Between July 2019 and February 2021, 60 patients were recruited in the trial. Eventually, fifty-two (86.7%) patients underwent D2 radical gastrectomy with evaluable pathological tumor response. Five of the 52 patients (9.6%) achieved a pathological complete response (pCR) and 11 (21.2%) patients experienced a near pCR (**Table S1**). The results showed that the combination of camrelizumab and mFOLFOX6 as neoadjuvant therapy for locally advanced GC/GEJ adenocarcinoma cancer significantly enhanced the anti-tumor effect. We obtained tumor tissues from 5 patients pre- and on-treatment neoadjuvant therapy. All patients completed 4 cycles of camrelizumab (200mg ivgtt on day1, q2w) plus mFOLFOX6 (oxaliplatin 85mg/m^2^ ivgtt, calcium levofolinate 200mg/m^2^ ivgtt, 5-Fu 400mg/m^2^ iv followed by 2.4mg/m^2^ CIV 46 hours on day 1, q2w) and underwent gastrectomy with D2 dissection. All of them gained R0 resection (100%), 1 patient (20%) achieved pCR, and 1 patient (20%) reached tumor pathology regression grade (TRG) 1. The patient that experienced pCR was HER-2 positive, the remaining four patients were HER-2 negative. Expression levels of EBV were negative for all. Clinical characteristics of the five patients are shown in **Table 1**.

**Table 1:** Patients’ clinical characteristics of scRNA-seq (N=5)*.

Characteristics	N	%
Sex		
male	4	80%
female	1	20%
ECOG (PS)		
0	2	40%
1	3	60%
Clinical stage		
T≥3	4	80%
N≥1	5	100%
Molecular-biological index (prior treatment)		
HER-2 postive	1	20%
MMR deficient	0	0%
Postoperative pathology		
Tumor pathology regression grade (TRG)^#^		
0	1	20%
1	1	20%
2	2	40%
3	1	20%
T stage		
0	1	20%
1	2	40%
2	0	0%
3	2	40%
N stage		
0	4	80%
3b	1	20%

*Neoadjuvant therapy: mFOLFOX6 (oxalipltain 85mg/m2 ivgtt, LV 200mg/m2 ivgtt,5-FU 400mg/m2 iv followed by 2.4mg/m2 civ 46h hours on day 1, q2w) plus Camrelizumab (200mg ivgtt on day 1, q2w).

#**TRG0**: No viable cancer cells, including lymph nodes.

**TRG1**: Single cells or rare small groups of cancer cells.

**TRG2**: Residual cancer cells with evident tumor regression but more than single cells or rate small groups of cancer cells.

**TRG3**: Extensive residual cancer with no evident tumor regression.

**Figure 1:**
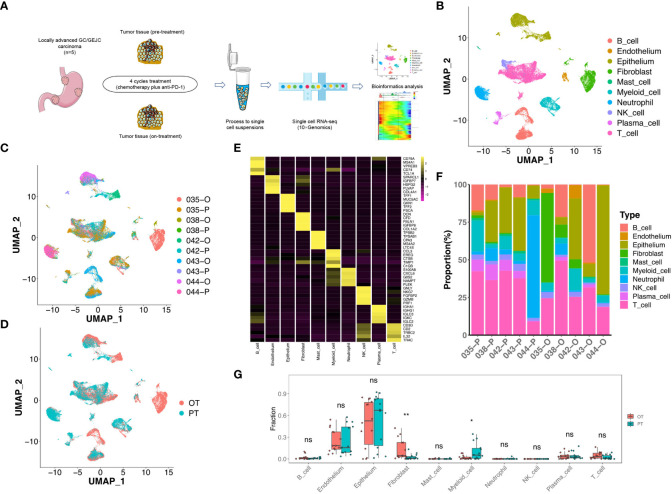
scRNA-seq profiling of the GC microenvironment during the combined therapy. **(A)** Schematic plot of the experimental strategy for 10× Genomics scRNA-seq. Ten site-matched endoscopic and surgical tumor samples were collected from 5 GC patients before and after 4 cycles treatment. **(B)** The two-dimensional UMAP plot showed the annotation and color codes for 10 major cell types in the GC ecosystem. **(C)** UMAP plot showed cell origins by patients and samples, the numbers represented patient ID, P represented “pre-treatment”, O represented “on-treatment”. **(D)** UMAP plot displayed cell origins by pre-treatment (PT) and on-treatment (OT). **(E)** Heatmap displayed the expression of marker genes of 10 major cell types. **(F)** Histogram indicated the proportion of major cell typesin each samples. **(G)** The cell proportion analysis of bulk samples in paired PT and OT tumors based on scRNA-seq data by CIBERSORTx, a paired two-sided Student’s t-test was used to assess the difference of cell infiltration. * p < 0.05; ** p < 0.01; ns, not significant.

The site-matched tumor tissues of pre- and on-treatment were subjected to scRNA-seq using 10× Genomics Chromium platform (**Figure 1A**). After quality control and filtering, we obtained 35,884 cells for further biological analysis (893-6,122 cells per sample), which generated ~334 million total mapped reads and 1,573 detected genes per cell on average.

To define TME cell populations of our GC cohort, we performed principal component analysis to evaluate variably expressed genes and subsequently used graph-based clustering to classify all cells. We identified and visualized 10 main cell types in paired pre-treatment (PT) and on-treatment (OT) samples using the Uniform Manifold Approximation and Projection (UMAP) method (**Figures 1B-D**). Non-immune cells (n = 11,090, 30.9%) primarily consisted of epithelium (6,157 cells, 17.2%, marked by EPCAM, KRT8 and KRT18), endothelium (634 cells, 1.8%, marked by PECAM1, ENG and VWF), and fibroblast (4,299 cells, 12.0%, marked by THY1, COL1A1 and COL1A2) cells based on the known markers (**Figure 1E**, **Figure S1**). Higher proportions of epithelial cells were observed compared to endothelial cells or fibroblasts in most samples (**Figure 1F**).

The identified immune cells (n = 24,794, 69.1%) contained natural killer (NK) cells (1,000 cells, 2.8%, marked by NKG7, GNLY and KLRF1), T cells (11,086 cells, 30.9%, marked by CD2, CD3D and CD3E), B cells (4,930 cells, 13.7%, marked by MS4A1 and CD79A), myeloid cells (2,976 cells, 8.3%, marked by CD14 and CD68), mast cells (295 cells, 0.8%, marked by TPSAB1 and CPA3), plasma cells (1,018 cells, 2.8%, marked by SDC1 and IGKC) and neutrophils (3,489 cells, 9.7%, marked by BASP1 and NAMPT) (**Figure 1E**, **Figure S1**). The infiltration levels of T cells was relatively higher compared to other immune cells in most PT and OT samples (**Figure 1F**). All immune cells showed infiltration variances before or during the treatment, which revealed the cellular heterogeneity among GC patients. Despite this variability, samples shared the same immune and non-immune cell types following treatment (**Figure 1F**). Considering that the sample size of our scRNA-seq cohort was limited, we performed a deconvolution algorithm CIBERSORTx to simulate the cell-type-specific gene expression profiles and predict the abundance of each cell type revealed by current scRNA-seq in our bulk RNA-seq dataset. We found that epithelial cells had higher proportions in both PT and OT samples, whereas myeloid cells and fibroblasts showed significant changes in OT compared with PT samples (**Figure 1G**). The bulk results indicated a degree of consistency in cell proportions within the scRNA-seq resullts.

### An interferon-γ signature of CD8^+^ T cells predicted effective response to the neoadjuvant therapy

**Figure 2:**
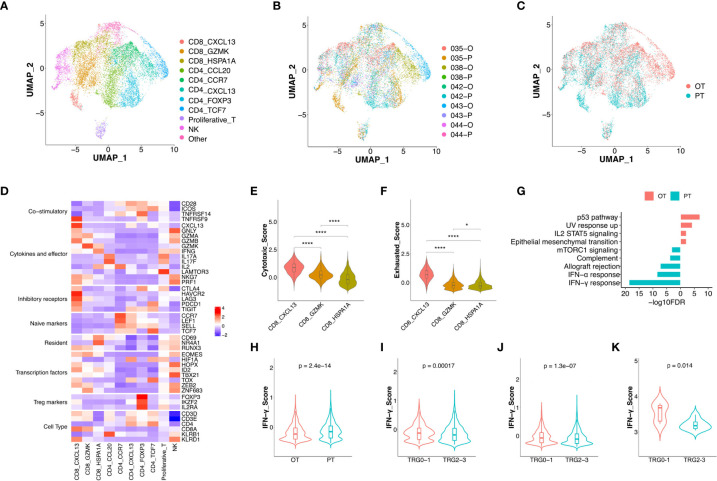
The changes of expression signatures in CD8^+^ T cells during the therapy. **(A)** UMAP plot showed the re-clustered T/NK cells labeled in different colors. **(B, C)** UMAP plot showed cell origins by samples **(B)** or the course of treatment **(C)**. **(D)** Heatmap indicated the expression of selected gene sets in T/NK subtypes, including naive, resident, inhibitory, cytokines, co-stimulatory, transcriptional factors, Treg markers and cell type. **(E, F)** Violin plot indicated the cytotoxic **(E)** and exhausted **(F)** scores in three subtypes of CD8^+^ T cell, significance was determined by unpaired Wilcoxon test. * p < 0.05; **** p < 0.001. **(G)** Pathway enrichment results of DEGs from CD8^+^ T cells between PTs and OTs. **(H)** The IFN-γ score of CD8^+^ T cells between PTs and OTs. **(I, J)** The IFN-γ signature of CD8^+^ T cells **(I)** or whole T cells **(J)** in baseline between TRG0-1 and TRG2-3 group. **(K)** The IFN-γ signature of bulk samples in PTs between TRG0-1 and TRG2-3 group. Significance of IFN-γ signature between two groups was determined by paired Wilcoxon test.

T cells (n = 11,086) represented the most prevalent immune cell type in the GC patients’ tumors. Further clustering of these T/NK cells revealed 11 sub-populations, including three clusters of CD8^+^ T cells (CD8 CXCL13, CD8 GZMK and CD8 HSPA1A), five clusters of CD4^+^ T cells (CD4 CCL20, CD4 CCR7, CD4 CXCL13, CD4 FOXP3 and CD4 TCF7), proliferative T cells, and NK cells (**Figure 2A**, **Figure S2A**). Although most subtypes of T cells were shared between PT and OT samples, CD4 CXCL13 and CD4 TCF7 cells were mainly enriched in OT samples, while CD8 CXCL13 were enriched in PT samples (**Figures 2B, C**).

Among three subtypes of CD8^+^ T cells, the CD8 CXCL13 cells showed high expression of inhibitory receptors (CTLA4, PDCD1 and TIGIT) and cytokines/effectors (CXCL13, GNLY and GZMA), suggesting that cytotoxic and exhausted states coexisted in the CD8 CXCL13 population (**Figure 2D**). The CD8 CXCL13 cells had significantly higher scores of cytotoxic and exhausted signatures than CD8 GZMK and CD8 HSPA1A cells (**Figures 2E, F**). We then analyzed differentially expressed genes (DEGs) and pathway enrichment of CD8^+^ T cells between PT and OT samples. Results revealed that up-regulated genes within OT samples were enriched in p53 and IL2 STAT5 pathways, while PT sample up-regulated genes were related to an IFN-γ/α response (**Figure 2G**, **S2B**). Accordingly, we found that CD8^+^ T cells of PT samples had higher expression of IFN-γ compared with CD8^+^ T cells in OTs (**Figure 2H**). Interestingly, patients of TRG0-1 group had a significantly higher IFN-γ score in CD8^+^ T cells or all T cell subtypes within baseline samples compared to the TRG2-3 group (**Figures 2I, J**). More importantly, the bulk sequencing data showed consistent results in the IFN-γ signature between TRG0-1 and TRG2-3 group (**Figure 2K**). In addition to the IFN-γ signature, we also found that the TRG0-1 group had a higher expression score of expanded immune cells in CD8^+^ T cells or all T cells of PT samples compared to TRG2-3 group (**Figures S2C, D**). These results suggest that higher scores of IFN-γ or an expanded immune signature predicted a better response to the combined therapy.

Among the five subtypes of CD4^+^ T cells, the CD4 CCL20 cells showed a relatively higher cytotoxic score, while CD4 CCL20 and CD4 CCR7 had a lower exhausted signature. In addition, an immunecheckpoint signature was up-regulated in CD4 CXCL13, CD4 FOXP3, and CD4 TCF7 cells (**Figure S2E-G**). Compared to OT samples, all CD4^+^ T cell subtypes in PT samples showed up-regulation of TNF-α signaling *via* NF-κB and an IFN-γ/α response (**Figure S2H**).

### Trajectory analysis uncovered dynamic changes in CD8^+^ T cells during treatment

**Figure 3:**
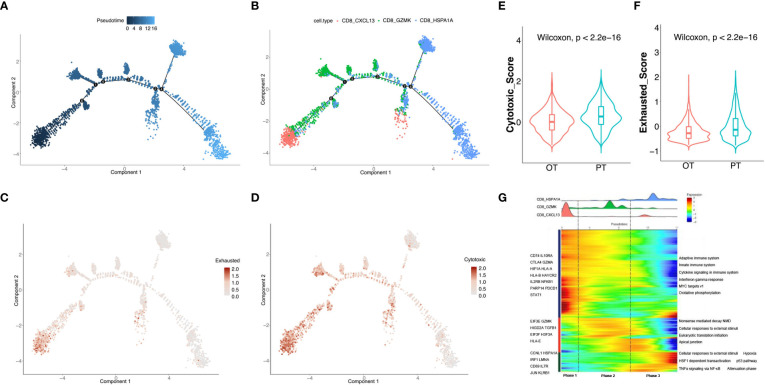
Analysis of the dynamic changes of CD8^+^ T cells during the treatment. **(A)** Monocle 2 trajectory analysis of CD8^+^ T cells labeled by pseudotime. **(B)** The trajectory analysis of CD8^+^ T cells labeled by CD8^+^ T cell subtypes. **(C, D)** Pseudotime plot showed the dynamics of exhausted **(C)** and cytotoxic signatures **(D)** in CD8^+^ T cells from PT and OT samples. **(E, F)** The cytotoxic **(E)** and exhausted scores **(F)** of CD8^+^ T cells displayed by violin plot between PTs and OTs, statistical analyses were paired Wilcoxon rank-sum test. **(G)** Heatmap indicated the dynamic changes in gene expression along the pseudotime (lower panel). The distribution of CD8^+^ T cell subtypes was divided into 3 phases along with the pseudotime. CD8^+^ T cell subtypes are labeled by colors (upper panel).

To uncover dynamic functional changes of CD8^+^ T cells in PT and OT samples, we adopted the Monocle 2 algorithm to chronologically order CD8^+^ T cells in pseudotime and indicate their trajectories (**Figure 3A**). The results showed that the trajectory path began with the CD8 CXCL13 cells, followed by the CD8 GZMK cells, and ended with CD8 HSPA1A cells (**Figure 3B**). We observed that CD8 CXCL13 cells were mainly found in PT samples harboring both cytotoxic and exhausted signatures, while more CD8 HSPA1A cells were aggregated in OT samples with low cytotoxic or exhausted signatures. Moreover, CD8 GZMK cells were enriched in both PT and OT samples, which appeared to be an intermediate state between cytotoxic and exhausted signatures (**Figures 2E, F**). Accordingly, the exhausted signature of CD8^+^ T cells decreased (**Figure 3C**), whereas the cytotoxic signature, despite an ultimate decrease, had a relatively higher score compared to the exhausted signature along the trajectory (**Figure 3D**). Indeed, the cytotoxic and exhausted scores of CD8^+^ T cells in PT samples were significantly higher thanOT samples (**Figures 3E, F**). These data indicate a coincidence of the pseudotime and the time course of changes in CD8^+^ T cells during treatment. However, we did not identify this coincidence in CD4^+^ T cells by pseudotime analysis.

We next investigated the transcriptional changes of CD8^+^ T cell associated with trajectory and found that the three clusters of CD8^+^ T cells could be categorized into 3 phases (**Figure 3G**). CD8 CXCL13 cells were primarily found in phase 1 and characterized by up-regulated expression of CTLA4, GZMA, HLA-A and PDCD1. Pathway enrichment analysis showed that MYC targets, the IFNγ response, and cytokine signaling were enriched in phase 1 (**Figure 3G**). Phase 2 had the most CD8 GZMK cells expressing *EIF3E*, *GZMK* and *TGFB1*, and genes involved in apical junctions and cellular responses to external stimuli (**Figure 3G**). CD8 HSPA1A cells were mostly found in phase 3 with high expression of CD69, JUN, KLRB1, and proteins enriched in TNFα signaling *via* NF-κB and p53 pathways.

### Intra-tumoral transcriptional response of epithelial cells to the combined therapy

**Figure 4:**
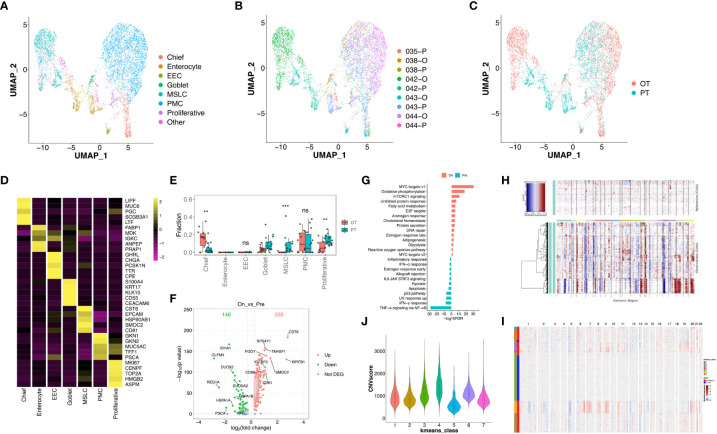
Transcriptional changes of epithelium in response to the treatment in GC ecosystem. **(A)** UMAP plot showed the re-clustered epithelium subtypes, EEC, enteroendocrine cell; MSLC, metaplastic stem-like cell; PMC, pit mucous cell. **(B, C)** UMAP plot showed epithelium origins by samples **(B)** or the course of treatment **(C)**. **(D)** Heatmap exhibited the expression of top markers in 7 subtypes of epithelium. **(E)** The cell proportion analysis of epithelium subtypes in bulk samples between PTs and OTs by CIBERSORTx. **(F)** Volcano plot exhibited DEGs of epithelium in comparison of PTs and OTs. **(G)** The GSEA results of DEGs in epithelium between PTs and OTs. **(H)** The inferred CNV results of epithelium (lower panel) compared with T cells (upper panel). **(I)** The k-means clustering of epithelium and selected T cells based on the inferCNV results, all cells could be divided into 7 classes. **(J)** Violin plot exhibited the CNVscores of 7 classes identified by k-means clustering. * p<0.05, ** p<0.01, *** p<0.001, ns, not significant.

We re-clustered 6,157 epithelial cells and found 7 subtypes in GC tumors (**Figures 4A-C**), including chief cells (marked by PGC and LIPF), enterocytes (FABP1 and ANPEP), enteroendocrine cells (CHGA and TPH1), goblet cells (MUC2), metaplastic stem-like cells (EPHB2 and SOX9), pit mucous cells (MUC5AC and TFF1) and proliferative cells (MKI67 and BIRC5, **Figure 4D**, **Figure S3**). Most subtypes were shared between PT and OT samples except for enterocytes which were enriched in PT tumors (patient 042 and 043, **Figures 4A-C**). This suggests that the combined therapy effectively reduced the proportion of enterocytes. The CIBERSORTx analysis of all cell subtypes identified that goblet, metaplastic stem-like, and proliferative cells were significantly reduced, and chief cells were significantly increased in bulk samples after therapy (**Figure 4E**). To reveal transcriptional changes within epithelial cells in response to therapy, we analyzed DEGs in PT and OT samples and found 558 genes were up-regulated after therapy, including *CST6*, *BPIFB1* and *SMOC2* (**Figure 4F**). Pathway analysis revealed up-regulation of MYC targets and oxidative phosphorylation and down-regulation of TNF-α signaling *via* NF-κB and IFN-γ response after therapy (**Figure 4G**).

Additionally, we distinguished malignant and non-malignant epithelial cells by inferring large-scale chromosomal copy-number variations (CNVs) in single epithelial cells (27, 30). Results revealed most epithelial cells exhibit relatively similars copy-number gain or losses compared to randomly selected T cells (**Figure 4H**). The k-means analysis showed that few epithelial cells were clustered with T cells in class 5 which had a lower CNV score (**Figures 4I, J**), suggesting that tumor-derived epithelial cells were mainly enriched in these samples.

### Intra-tumoral transcriptional response of fibroblast to the combined therapy

**Figure 5:**
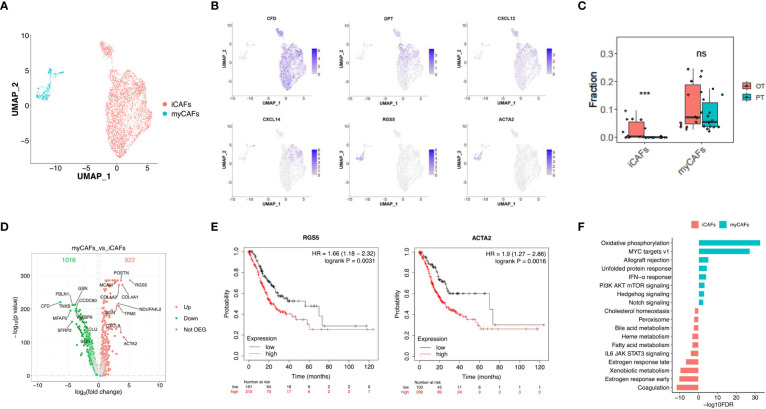
Two distinct subtypes of CAFs showed transcriptional differences in GC ecosystem. **(A)** UMAP plot showed the re-clustered fibroblast subtypes, iCAFs and myCAFs. **(B)** Feature plot displayed the expression levels of markers in iCAFs and myCAFs. **(C)** The cell proportion analysis of fibroblast subtypes in bulk samples between PTs and OTs by CIBERSORTx. **(D)** Volcano plot exhibited DEGs of myCAFs compared with iCAFs. **(E)** The survival analysis of mRNA expression of RGS5 and ACTA2 in TCGA GC cohort. **(F)** GSEA results showed the Hallmark gene sets enriched in iCAFs or myCAFs. ***p<0.001.

Cancer-associated fibroblasts (CAFs) (n = 4,299) showed two distinct subtypes with unique gene signatures upon re-clustering analysis. Common fibroblast markers such as COL1A1 and THY1 were extensively expressed in both sub-populations, confirming their fibroblast identity (**Figure 5A**, **Figure S4A**). Sub-cluster 1 of fibroblasts appeared in OT sampels with strong expression of CFD, DPT and various chemokines, including CXCL12 and CXCL14 (**Figure 5B**, **Figures S4B, C**). This signature is similar to that of inflammatory CAFs (iCAFs) described in pancreatic cancer (31, 32). Sub-cluster 2 distributed between PT and OT samples had high expression of RGS5 and ACTA2, which is similar to myofibroblastic CAFs (myCAFs) (**Figure 5B**, **S4B, C**). These results demonstrate that CAF populations within GC have similar subtypes to other cancers (32, 33). The CIBERSORTx analysis also validated that iCAFs are significantly increased in OT samples, while myCAFs had no obvious change between PT and OT samples (**Figure 5C**). Our analysis also identified abundant DEGs for myCAFs and iCAFs (**Figure 5D**). Up-regulated RGS5 and ACTA2 expression in myCAFs indicated poor overall survival of GC patients in TCGA and GEO datasets (**Figures 5E**, **S4D**). Coagulation and estrogen response pathways were significantly up-regulated in iCAFs, while myCAFs had upregulated oxidative phosphorylation and MYC targets v1 (**Figure 5F**).

### Intra-tumoral transcriptional response of B and myeloid cells to the combined therapy

**Figure 6:**
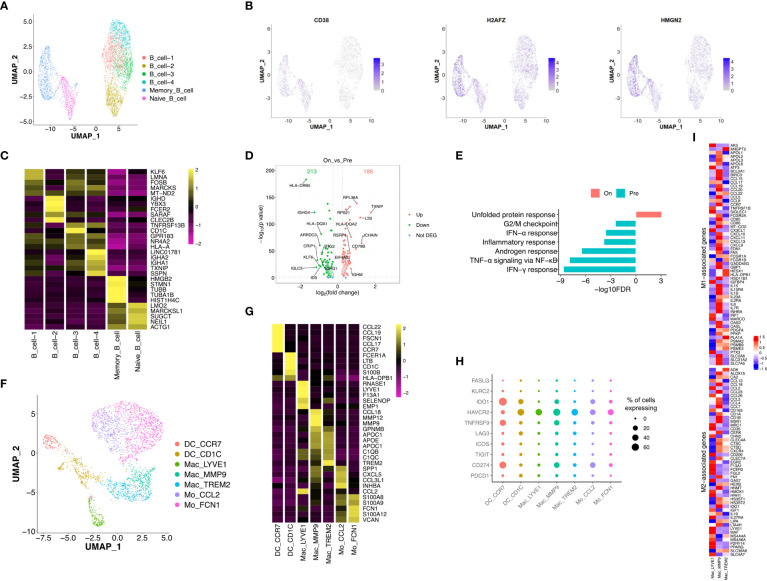
Transcriptional diversity of B and myeloid cells in response to the treatment. **(A)** UMAP plot showed the re-clustered subtypes of B cells. **(B)** Feature plot displayed the expression of markers in memory/naïve B cells. **(C)** Heatmap exhibited the expression of top markers in 6 subtypes of B cells. **(D)** Volcano plot exhibited DEGs of B cell-1/2/3/4 in comparison of PTs and OTs. **(E)** The GSEA results of above DEGs in B cells between PTs and OTs. **(F)** UMAP plot showed the re-clustered subtypes of myeloid cells, DC, dendritic cell; Mac, macrophage; Mo, monocyte. **(G)** Heatmap indicated the expression of marker genes in myeloid subtypes. **(H)** Dotplot showed the percentage of cells in each subtype of myeloid cells expressing immune checkpoint and evasion genes. **(I)** Heatmap of normalized expression for curated M1- and M2-associated genes within macrophage subtypes.

Re-clustering of B cells (n = 4,930) showed 6 subtypes with distinct distributions, including memory and naïve B cells in OT samples and 4 sub-populations of B cells in PT and OT samples (**Figures 6A**, **S5A, B**). Memory B cells displayed high expression of HMGN2 and H2AFZ (**Figure 6B**), naïve B cells up-regulated CD38 (**Figure 6B**), and other B cell clusters showed distinct expression signatures (**Figure 6C**). We observed that patient 043 had high infiltration of B cells, both memory and naïve, in response to the combined therapy (**Figure S5A**). Therefore, we analyzed DEGs in the other four subtypes of B cells between PT and OT samples and found many genes showed differential expression in response to the combined therapy (**Figure 6D**). Similar to the T and epithelial cells,IFN response and TNF-α signaling *via* NF-κB were enriched in B cells from PT samples (**Figure 6E**).

Next, we analyzed innate immune cell populations and their role tumor progression and response to immunotherapy. We extracted myeloid cells (n = 2,976) *via* CD14 and CD68 expression, and re-clustered them into two subtypes of monocytes (Mo CCL2 and Mo FCN1), two subtypes of dendritic cells (DC CCR7 and DC CD1C), and three subtypes of macrophages (Mac LYVE1, Mac MMP9 and Mac TREM2) (**Figure 6F**). Most subtypes of myeloid cells could be found in both PT and OT samples, while Mac LYVE1 and Mac MMP9 showed specific enrichment (**Figures S5C, D**). In addition, these subtypes of myeloid cells differentially expressed marker genes and immunomodulatory genes (**Figure 6G, H**).

Twoforms of macrophage polarization are M1 (or classic) and M2 (or alternative) and are characterized by antitumor- responses or suppression, respectively (24). We found Mac LYVE1 highly expressed a set of M2-associated genes, while the other two subtypes did not show tendentious expression (**Figure 6I**). Many chemokines were differentially expressed among macrophages, including M1 markers CCL5, M2 markers CCL13 and CCL18 (**Figure 6I**), as previously described in GC (14, 34).

### Complex cell–cell interactions in GC TME during the neoadjuvant therapy

To uncover changes in cell-cell communication during treatment, we used the CellPhoneDB to identify ligand–receptor pairs and molecular interactions among major cell populations.

**Figure 7:**
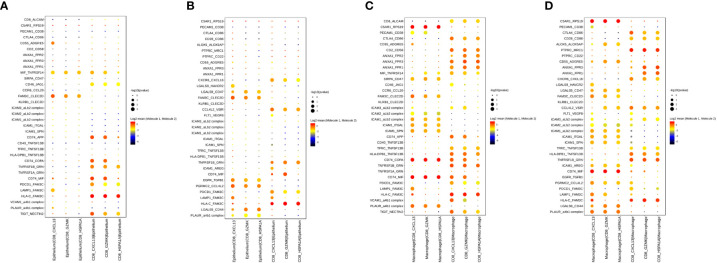
Cell–cell interactions of CD8^+^ T cell and epithelium/macrophage during the combined treatment in GC ecosystem. **(A, B)** Bubble plots showed the selected ligand-receptor interactions between CD8^+^ T cells and epithelium in PTs **(A)** and OTs **(B)**. **(C, D)** Bubble plots showed the selected ligand-receptor interactions between CD8^+^ T cells and macrophages in PTs **(C)** and OTs **(D)**. P values were indicated by circle size with permutation test. The log-transformed means of the average expression levels of interacting molecule 1 in cluster 1 and interacting molecule 2 in cluster 2 were indicated by color.

Considering the significant role CD8^+^ T cells in immunotherapy response, we explored interactions between epithelial cells and three CD8^+^ T cell subtypes in the TME of PT and OT samples. Of note, CD74 and the corresponding receptors (APP, COPA and MIF) were significantly expressed in epithelial and CD8^+^ T cells of PT samples (**Figure 7A**). In OT samples, CXCR6-CXCL16 and EGFR-TGFB1 pairs were newly identified, and CD74 receptors were decreased between epithelial and CD8^+^ T cells (**Figure 7B**). This suggests that communication between tumor and CD8^+^ T cells is remodeled by combined therapy in GC.

Additionally, we identified increased communication between macrophages and CD8^+^ T cells (**Figures 7C, D**). Notably, ANXA1-FPR1/2/3 and ICAM proteins were widely expressed and mediated the interactions between macrophages and CD8^+^ T cells in both PT and OT samples (**Figures 7C, D**). These results indicate that the anti-inflammatory and intercellular adhesion interactions remained stable during the treatment.

## Discussion

ICB combined with chemotherapy has been shown to improve survival for advanced GC patients (35, 36). More specifically, our research revealed the combination of anti-PD-1 antibody and chemotherapy achieved better pCR and R0 resection rates compared to the standard neoadjuvant chemotherapy. Although immunotherapy can induce robust and durable responses, the response only occurs in a minority of patients. Establishment of predictive biomarkers for immunotherapy is important to maximize therapeutic benefits. In the present study, we comprehensively assessed intratumoral transcriptomic changes at the single-cell level in GC patients receiving neoadjuvant ICB. Although several studies have characterized treatment-naïve microenvironment heterogeneity with single-cell resolution in GC (13–15, 17), our study is the first to report on exploring how the combined immunotherapy and chemotherapy affects expression programs of immune and non-immune cells in the GC microenvironment.

In this study, we found that a high IFN-γ signature within CD8^+^ T cells had predictive value in the therapeutic outcome of neoadjuvant ICB. Ayers et al. identified immune-related signatures that correlate to clinical outcome using bulk tumor samples in different clinical studies of pembrolizumab. This work started with a small pilot study of melanoma and eventually defined a pan-tumor T cell–inflamed gene expression profile in 9 cancers, including GC (23). Our study confirmed that an IFN-γ signature of 6 genes and an expanded immune signature of 18 genes in T cells could predict the efficacy of the combined anti-PD-1 and chemotherapy. Moreover, the scRNA-seq and bulk RNA-seq showed consistent results, which confirms that the IFN-γ or immune-related signatures can guide clinical prediction in future neoadjuvant ICB in GC. However, we need more samples to validate the IFN-γ signature by scRNA-seq and further elucidate the mechanism about the changes of IFN-γ signature pre and post neoadjuvant therapy.

CAFs are critical components of the tumor microenvironment with both pro- and anti-tumorigenic effects in a context-dependent manner (37, 38). The heterogeneity of CAFs have been well described in other cancers by scRNA-seq, including ovarian cancer (39), prostate cancer (10) and intrahepatic cholangiocarcinoma (40). However, the diversity of CAFs in GC is rarely reported. In our GC cohort, we found two CAF subpopulations iCAF and myCAF, with different transcriptional signatures. Jeong et al. also found distinct sub-clusters of fibroblasts in diffuse-type GC. Among them, the Fibro2 cells were identified as myofibroblasts and the Fibro1 possessed immune-mediated inflammatory features with enhanced interferon signaling (41). These results confirmed the heterogeneity of CAFs in GC. However, our data showed that iCAFs were enriched in OT samples with different TRG (one was complete tumor regression and the other was TRG3), which does not support the association of iCAFs with treatment efficacy of neoadjuvant ICB. However, an increased sample size is needed to verify the relationship between CAFs and therapeutic effect in GC.

Finally, we identified tumor-associated macrophages (TAMs) as a heterogenous immune cell population that had increased interactions with CD8^+^ T cells. The plural functions of TAMs and their roles in immunotherapy have been extensively reviewed (42, 43). Our work revealedmany cell–cell interactions, such as intercellular adhesion proteins, between TAMs and CD8^+^ T cells were insusceptible to the neoadjuvant ICB, suggesting additional reciprocal regulation between TAMs and CD8^+^ T cells warrant further functional experimentation.

## Conclusions

In conclusion, our study used scRNA-seq to comprehensively identify the dynamic landscape of the intra-tumoral cellular transcriptome in GC between pre- and on-treatment with neoadjuvant immunotherapy combined chemotherapy. This work revealed a IFN-γ signature of CD8^+^ T cells may possess potential value in predicting response to the combined therapy in GC patients.

## Data Availability

The data presented in the study are deposited in the Genome Sequence Archive (GSA) repository (URL: https://ngdc.cncb.ac.cn/gsa-human/). The accession number: HRA003148.

## Supplementary material

Supplementary Figure 1 Feature plot displayed the expression of markers in 10 major cell types.

Supplementary Figure 2 The expression signatures of CD8^+^ and CD4^+^ T cells during the therapy. **(A)** Heatmap indicated the expression of marker genes of T/NK cell subtypes. **(B)** Volcano plot exhibited DEGs of CD8^+^ T cells in comparison of PTs and OTs. **(C, D)** The expanded immune scores of CD8^+^ T cells **(C)** or whole T cells **(D)** in baseline between TRG0-1 and TRG2-3 group. **(E-G)** Violin plot indicated the cytotoxic **(E)**, exhausted **(F)** and immune **(G)** scores in five subtypes of CD4^+^ T cell, all statistical analyses were Kruskal-Wallis test. (H) Pathway enrichment results of DEGs from CD4^+^ T cells between PTs and OTs.

Supplementary Figure 3 Feature plot displayed the expression of markers in epithelial subtypes.

Supplementary Figure 4 Two subtypes of CAFs in GC ecosystem. **(A)** Feature plot displayed the expression of markers of CAFs in two subtypes. **(B, C)** UMAP plot showed cell origins by samples (B) or the course of treatment **(C)**. **(D)** The survival analysis of mRNA expression of RGS5 and ACTA2 in GEO GC cohorts.

Supplementary Figure 5 The re-clustering analysis of B and myeloid cells. **(A, B)** UMAP plot showed the origins of B cells by samples **(A)** or the course of treatment **(B)**. **(C, D)** UMAP plot showed the origins of myeloid cells by samples **(C)** or the course of treatment **(D)**.
