# Supplementary figures and images for: A high interferon gamma signature of CD8+ T cells predicts response to neoadjuvant immunotherapy plus chemotherapy in gastric cancer
Source: Front Immunol. 2023 Jan 5;13:1056144. doi: 10.3389/fimmu.2022.1056144 (PMC9849934; doi:10.3389/fimmu.2022.1056144)

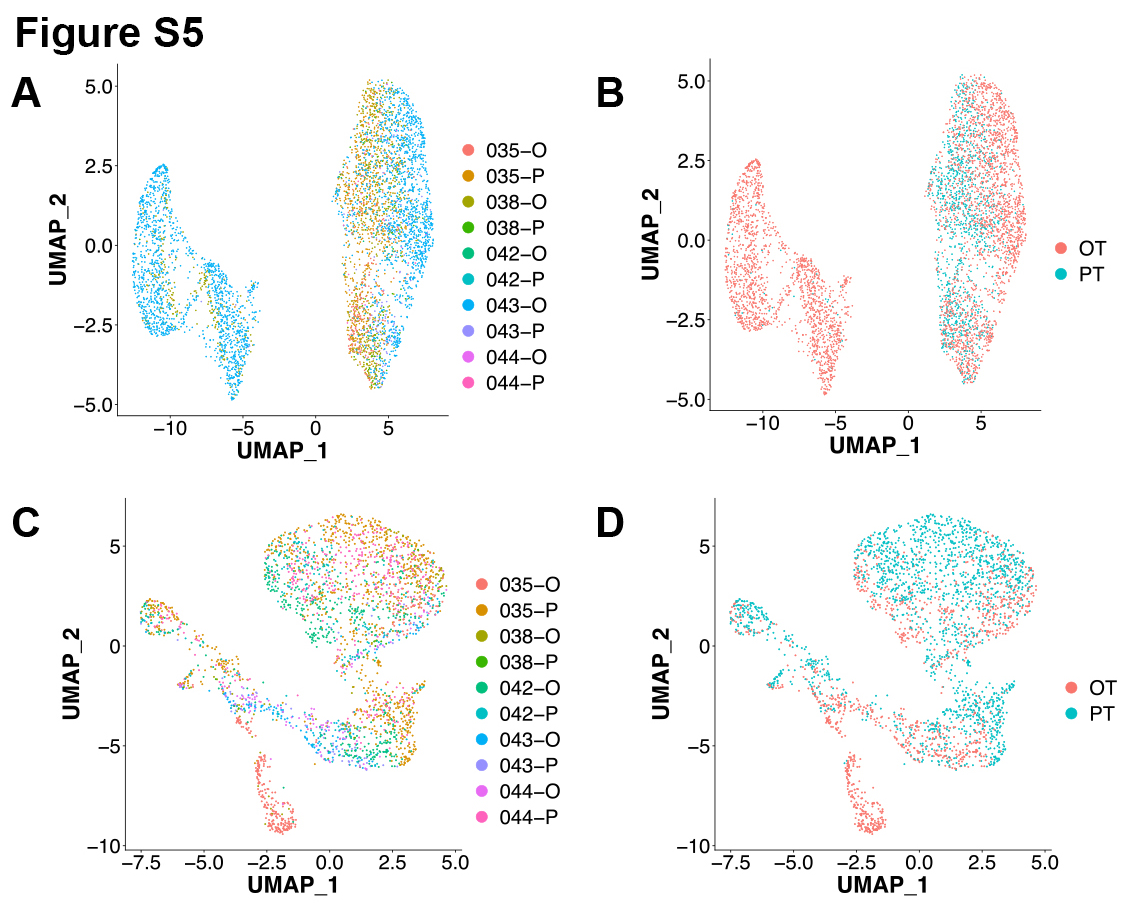

Supplement: Supplementary Figure 5 — The re-clustering analysis of B and myeloid cells. (A, B) UMAP plot showed the origins of B cells by samples (A) or the course of treatment (B). (C, D) UMAP plot showed the origins of myeloid cells by samples (C) or the course of treatment (D). [file Image_5.tif]
