# Supplementary material for: A high interferon gamma signature of CD8+ T cells predicts response to neoadjuvant immunotherapy plus chemotherapy in gastric cancer
Source: Front Immunol. 2023 Jan 5;13:1056144. doi: 10.3389/fimmu.2022.1056144 (PMC9849934; doi:10.3389/fimmu.2022.1056144)
Supplement: Supplementary file 6 [file Table_1.docx]

| **Table S1.Baseline Characteristics of Enrolled Patients(n=60)** | |
| --- | --- |
| **Variables** | Total (n = 60) |
| Median age, years (range) | 58 (29, 72) |
| **Sex, n (%)** |  |
| Male | 43 (71.7) |
| Female | 17 (28.3) |
| **ECOG performance score, n (%)** |  |
| 0 | 36 (60.0) |
| 1 | 24 (40.0) |
| **Primary tumor location, n (%)** |  |
| Gastro-esophageal junction | 38 (63.3) |
| Gastric | 22 (36.7) |
| **Clinical stage, n (%)** |  |
| II | 2 (3.3) |
| III | 58 (96.7) |
| **D2 radical gastrectomy** | 52(86.7%) |
|  |  |
